# Alström's Syndrome, Leber's Hereditary Optic Neuropathy, or Retinitis Pigmentosa? A Case of Misdiagnosis

**DOI:** 10.1155/2023/9409036

**Published:** 2023-11-16

**Authors:** Palaiologos Alexopoulos, Chrysanthos Symeonidis, Tryfon Rotsos

**Affiliations:** ^1^Department of Ophthalmology, NYU Langone Health, New York, NY, USA; ^2^1st Department of Ophthalmology, University of Athens, G. Gennimatas General Hospital, 154, Mesogion Av., 115 27 Athens, Greece

## Abstract

A case of a patient with the Alström syndrome (AS) that was misdiagnosed as Leber's hereditary optic neuropathy or retinitis pigmentosa for 13 years is presented. AS is a rare genetic disorder caused by mutations in the *ALMS1* gene. AS may lead to abnormal ciliary formation and function. AS affects metabolism, and symptomatology includes type 2 diabetes mellitus (*T2DM*), obesity, hypogonadism and gynecomastia in males, progressive bilateral sensorineural hearing loss, cardiomyopathy, nonalcoholic fatty liver disease (*NAFLD*), cirrhosis, and chronic progressive kidney disease. The onset of the above symptoms may vary significantly. The ophthalmic manifestation is early onset cone-rod dystrophy that starts as progressive vision loss, photophobia, and nystagmus in the first months of life. An accurate diagnosis may enable specialists to facilitate a significantly positive effect in the everyday life of a patient. Genetic counseling may also be recommended for these patients. Diagnosis was confirmed by DNA testing, thus highlighting its necessity in everyday practice.

## 1. Introduction

The Alström syndrome (AS), first reported in 1959, is an autosomal recessive genetic disorder caused by mutations in the *ALMS1* (Alström syndrome 1) gene [1]. Up to date, approximately 1,200 cases have been reported in the literature, with an incidence rate of 1/500,000 to 1/1,000,000 cases [2, 3].

AS is caused by mutations in the *ALMS1* gene, and as an autosomal recessive condition, both copies of the gene are required to be affected [2]. The *ALMS1* protein is found in cilia and is a major component of ciliary anatomy; therefore, AS is essentially a ciliopathy, as mutations in both *ALMS1* genes lead to abnormal ciliary formation and function [4]. It has also been shown to play a role in metabolism, cell differentiation, signaling pathways, cell cycle control, and intracellular trafficking [5].

AS constitutes a multisystem condition with variable presentation regarding severity, systems affected, and affected family members [6]. Symptoms include type 2 diabetes mellitus (*T2DM*) and obesity, hypogonadism and gynecomastia in males, progressive bilateral sensorineural hearing loss, cardiomyopathy, nonalcoholic fatty liver disease (*NAFLD*), cirrhosis, and chronic progressive kidney disease [2, 7]. A number of the aforementioned symptoms may appear early in life. The ophthalmic manifestation is early onset cone-rod dystrophy that manifests as progressive vision loss, photophobia, and nystagmus in the first months of life (up to 15 months of age).

No treatment is currently available for AS. Management should include detection and monitoring of its complications using primarily blood tests (e.g., glucose and biochemical panel profiling for *T2DM* and obesity and liver function tests for cirrhosis), urinalysis, and organ-specific tests (for example, electrocardiography and echocardiograms for cardiomyopathy and visual acuity for retinopathy) [8]. Genetic counseling is also highly recommended, since these patients pass on a mutated gene to their descendants as well as genetic testing of siblings and parents. Given its variable presentation, its prognosis is difficult to determine, though AS often leads to organ failure; life expectancy is less than 50 years [7].

## 2. Case Report

A 5-month-old female infant presented with horizontal and rotational nystagmus of pendular characteristics, occasionally rebounding, which disappeared with fixation on near objects. Nystagmus was noted to be more intense on the hyperopic left eye. Fundoscopic examination was normal bilaterally at the time. Electroencephalography was characterized by sharp theta waves, nystagmus episodes, and slow delta waves during head movements originating from the parietal and occipital lobes. Imaging of the brain with computed tomography (CT) and magnetic resonance (MRI) was normal.

Later in life, the patient was diagnosed with T2DM and obesity as well. Within a 5-year period (2016 to 2021), her metabolic profile, including vitamin B_12_, glucose profile (HbA1c, glucose tolerance tests), liver function tests, and complete blood count were normal except for vitamin D insufficiency (low levels of vitamin D_3_) at various time points. Abdominal ultrasound did not reveal abnormalities in the liver, common bile duct, portal vein, gallbladder, pancreas, spleen, or kidneys. Visual acuity was light perception in both eyes and had different diagnoses from different hospitals (Leber's hereditary optic neuropathy and retinitis pigmentosa).

**Figure 1:**
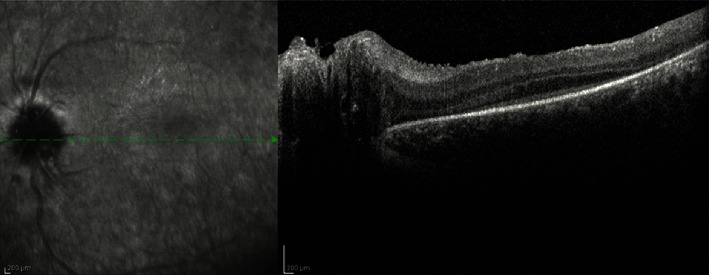
OCT scan of the left optic nerve head and macular region depicting disruption in the photoreceptor layer.

**Figure 2:**
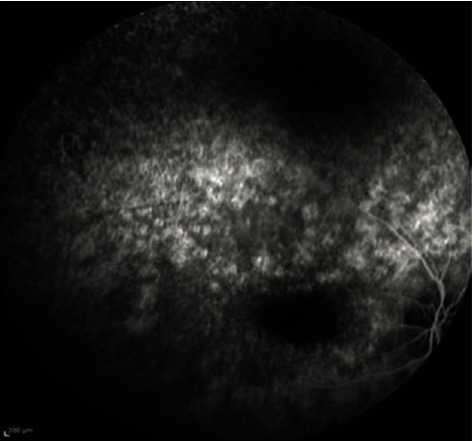
Fluorescein angiography with the vessel attenuation and the hyperfluorescent areas in the posterior pole and the periphery (window defects).

**Figure 3:**
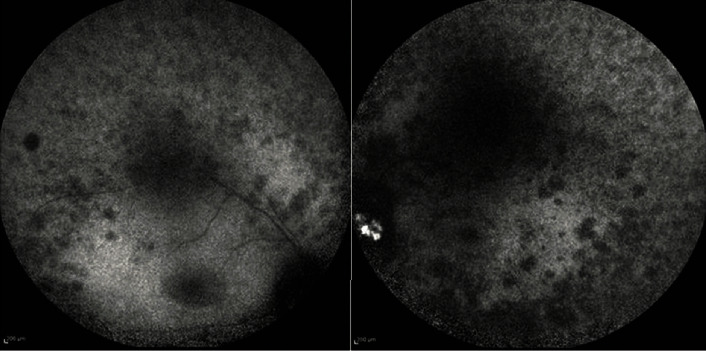
Fundus autofluorescence (FAF) in the right and left eyes with extended hyporeflective areas in the posterior pole and the periphery.

**Figure 4:**
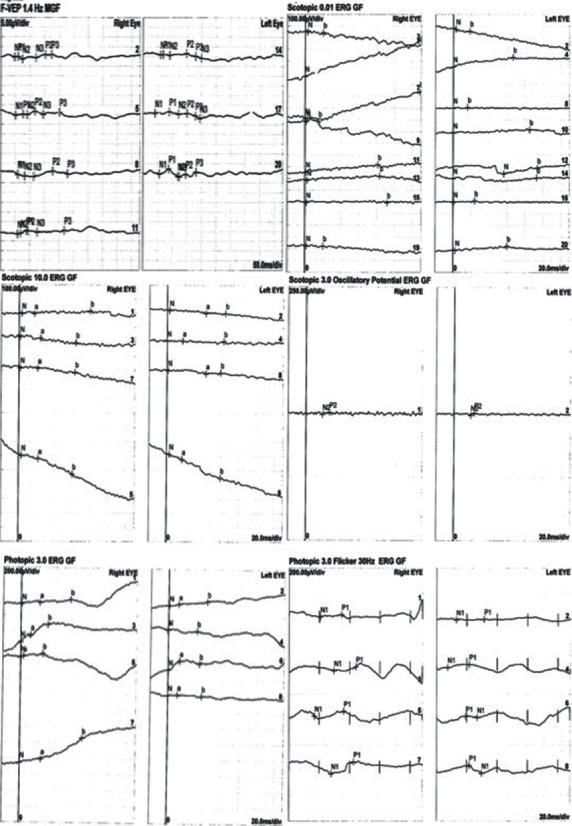
Electroretinogram (ERG, scotopic negative, photopic negative, and bright flash testing) and visual evoked potential (VEP) findings were abnormal, with the recordings noted not surpassing the electrical noise.

The patient presented in our department in 2021. She was 17 years old at the time. Best corrected visual acuity (BCVA) was light perception in both eyes. BCVA was light perception in both eyes reportedly since the age of 13. OCT scans of the macular region displayed significant photoreceptor layer disruption in both eyes with minimal preservation of this layer around the central fovea (Figure 1). Fluorescein angiography (FA) revealed vessel attenuation and hyperfluorescent areas in the posterior pole (window defects, Figure 2). Autofluorescence (FAF) revealed extended disruption of the retinal pigment epithelium (RPE) metabolic activity in the posterior pole and periphery OU (Figure 3). The findings on the electroretinogram (ERG, scotopic negative, photopic negative, and bright flash testing) and visual evoked potentials (VEP) were abnormal, with the recordings noted not surpassing electrical noise (Figure 4). Based on these findings, the diagnosis of early onset retinal dystrophy was established, with a recessive inheritance pattern. The presumed diagnosis at the time was Leber's hereditary optic neuropathy.

Following the initial diagnosis, genetic DNA testing established the diagnosis of the Alström syndrome. DNA was isolated from whole peripheral blood, and simultaneous molecular control of the complete coding sequence of 125 genes of known function involved in hereditary retinopathies by the Next Generation Sequencing method (Ophthalmic Genetic Unit, Athens) was performed.

Clinical diagnosis was potential congenital malformation according to Leber's hereditary optic neuropathy. Molecular analysis was positive for pathogenic mutations. The heterozygous genetic variants c.4156dup, p.(Thr1386Asnfs^∗^15) and c.(9542+1_9543-1)_(9784+1_9785-1) del involving exons 8 and 11, respectively, of the *ALMS1* gene (Alström's syndrome) were detected (MIM 606844). Mutations in the *ALMS1* gene are responsible for the Alström syndrome (MIM 203800).

The mutations identified in the genetic material of our patient are predicted to be pathogenic as they cause the premature termination of *ALMS1* protein synthesis (protein truncating mutations). One of them, c.4156dup, p.(Thr1386Asnfs^∗^15) has previously been reported in the international literature but with a different second mutation, while the second mutation reported here, which is recommended for the loss of exon 11 of the gene, has not been previously reported in similar published papers. Consequently, the combination of the two mutations has not been previously reported in the relevant literature.

The presence of the detected mutations c.4156dup, p.(Thr1386Asnfs^∗^15) and c.(9542+1_9543-1)_(9784+1_9785-1) del of the *ALMS1* gene in the genetic material of our patient is most likely the cause of her condition which according to her genetic diagnosis is the Alström syndrome, which belongs to the group of ciliopathies and is inherited with an autosomal recessive type of inheritance. It should be noted that the phenotypic manifestations of the Alström syndrome can appear at various times in a patient's life. They may also vary greatly both between families and between members of the same family [8].

All the children of our patient must necessarily be heterozygous carriers of one of the two detected *ALMS1* gene mutations. As carriers of an autosomal recessive hereditary disease mutation, these children will be asymptomatic, provided the father is not also a carrier of a pathogenic mutation of the same gene.

Our patient is additionally heterozygous for the genetic variant c.367>T, p.(Arg123Cys) of unclear clinical significance (*VUS*) in the *RP1* gene (*RP1* axonemal microtubule associated, MIM 603937). This genetic variant is registered in the international database of clinical genetic data ClinVar with the code 1025517 in the context of clinical control without clarification while it has not been reported in relation to the diseases associated with the *RP1* gene in the international literature. Mutations in the *RP1* gene are responsible for autosomal recessive and dominant retinitis pigmentosa 1 (retinitis pigmentosa 1, MIM 180100, AD, AR). The detected mutation is located in exon 2, and based on the available data, it would be associated with the recessive form of the condition for which the presence of two mutations is required. No second or potential pathogen was identified pathogenic mutation in the *RP1* gene in our patient. Moreover, there are 2 heterozygotes in the gnomAD international control population genetic databases (with more than 120,000 exomes and 15,000 genomes) which means that this genetic deletion cannot cause severe disease at least in childhood. Consequently, it is not considered possible that this genetic variant is associated with the clinical status of the patient, especially in the light of the identification of the two pathogenic mutations in the *ALMS1* gene.

## 3. Discussion

The two mutations detected were c.4156dup and c.(9542+1_9543-1)_(9784+1_9785-1) del of exons 8 and 11 of the *ALMS1* gene, respectively. In the gnomAD international genetic database, which contains over 120,000 exons and 15,000 genomes, only 6 heterozygotes are reported. The first genetic variation explained in this case has also been reported with different genetic variations, namely, c.3163dup in a patient with the Alström syndrome [9] and c.6436C>T in a toddler with dilated cardiomyopathy [10]. The second mutation of exon 11 has not been reported in the literature yet, and thus, this mutation combination has also not been previously identified.

Although there is no current treatment available for AS, accurate diagnosis is essential. The patient presented here was not correctly diagnosed for 13 years. She was given different diagnoses (e.g., Leber's hereditary optic neuropathy and retinitis pigmentosa) and was informed that no symptomatic treatment was available for her condition. The value of DNA testing is apparent in similar cases. An accurate diagnosis using appropriate tests (e.g., glucose values, biochemical panel profiling for T2DM and obesity, liver function tests for cirrhosis, urinalysis, electrocardiography, and echocardiogram for cardiomyopathy) may enable specialists to bring about a positive effect in the everyday life of a patient [11].

Genetic counseling may also be recommended for patients with the Alström syndrome. Diagnosis was confirmed by DNA testing, thus highlighting its necessity in similar cases in everyday practice.

## Data Availability

Relevant data is available on request. Please contact Dr. Chrysanthos Symeonidis (chrys2209@gmail.com).
